# A Multicomponent Communication Intervention to Reduce the Psycho-Emotional Effects of Critical Illness in ICU Patients Related to Their Level of Consciousness: CONECTEM

**DOI:** 10.3390/jcm15031154

**Published:** 2026-02-02

**Authors:** Marta Prats-Arimon, Montserrat Puig-Llobet, Mar Eseverri-Rovira, Elisabet Gallart, David Téllez-Velasco, Sara Shanchez-Balcells, Zaida Agüera, Khadija El Abidi-El Ghazouani, Teresa Lluch-Canut, Miguel Angel Hidalgo-Blanco, Mª Carmen Moreno-Arroyo

**Affiliations:** 1 Department of Clinical and Fundamental Nursing, Faculty of Nursing, University of Barcelona, C/Feixa Llarga s/n, 08870 Hospitalet de Llobregat, Spain; mprats22@ub.edu (M.P.-A.); miguelhidalgo@ub.edu (M.A.H.-B.); carmenmoreno@ub.edu (M.C.M.-A.); 2 Research Group in Mental Health, Psychosocial and Complex Nursing Care (NURSEARCH), Faculty of Nursing, University of Barcelona, C/Feixa Llarga s/n, 08870 Hospitalet de Llobregat, Spain; sara.sanchez@ub.edu (S.S.-B.); zaguera@ub.edu (Z.A.); kadi.elabidi@ub.edu (K.E.A.-E.G.); tlluch@ub.edu (T.L.-C.); 3 Department of Public Health, Mental Health and Maternal-Child Nursing, Faculty of Nursing, University of Barcelona, C/Feixa Llarga s/n, 08870 Hospitalet de Llobregat, Spain; 4 Intensive Care Unit, Vall d’Hebron University Hospital, Pg. de la Vall d’Hebron, 119, Horta-Guinardó, 08035 Barcelona, Spain; mmar.eseverri@vallhebron.cat (M.E.-R.); bet.gallart@vallhebron.cat (E.G.); david.tellez@vallhebron.cat (D.T.-V.); 5 CIBER Fisiopatología Obesidad y Nutrición (CIBERobn), Instituto de Salud Carlos III, 28029 Madrid, Spain

**Keywords:** augmentative alternative communication, nurse-patient interaction, phyco-emotional effects, critical care

## Abstract

**Background/Objectives:** Patients admitted to intensive care units (ICUs) are confronted with complex clinical situations that impact their physical condition and psychological well-being. Psycho-emotional disorders such as pain, anxiety and post-traumatic stress are highly prevalent in this context, significantly affecting both the patient’s experience and the quality of care provided. Effective communication can help manage patients’ psycho-emotional states and prevent post-ICU disorders. To evaluate the effectiveness of the CONECTEM communicative intervention in improving the psycho-emotional well-being of critically ill patients admitted to the intensive care unit, regarding pain, anxiety, and post-traumatic stress symptoms. **Methods**: A quasi-experimental study employed a pre–post-test design with both a control group and an intervention group. The study was conducted in two ICUs in a tertiary Hospital in Spain. A total of 111 critically ill patients and 180 nurse–patient interactions were included according to the inclusion/exclusion criteria. Interactions were classified according to the level of the patient’s consciousness into three groups: G1 (Glasgow 15), G2 (Glasgow 14–9), and G3 (Glasgow < 9). Depending on the patient’s communication difficulties, nurses selected one of three communication strategies of the CONECTEM intervention (AAC low teach, pictograms, magnetic board, and musicotherapy). Pain was assessed using the VAS or BPS scale, anxiety using the STAI, and symptoms of PTSD using the IES-R. The RASS scale was utilized to evaluate the degree of sedation and agitation in critically ill patients receiving mechanical ventilation. Data analysis was performed using repeated ANOVA measures for the pre–post-test, as well as Pearson’s correlation test and Mann–Whitney U or Kruskal–Wallis statistical tests. **Results**: The results showed pre–post differences consistent with pain after the intervention in patients with Glasgow scores of 15 (*p* < 0.001) and 14–9 (*p* < 0.001) and in anxiety (*p* = 0.010), reducing this symptom by 50% pre-test vs. 26.7% post-test. Patients in the intervention group with levels of consciousness (Glasgow 15–9) tended to decrease their post-traumatic stress symptoms, with reductions in the mean IES scale patients with a Glasgow score of 15 [24.7 (±15.20) vs. 22.5 (±14.11)] and for patients with a Glasgow score of 14–9 [(Glasgow 14–9) [30.2 (±13.56) 27.9 (±11.14)], though this was not significant. Given that patients with a Glasgow score below 9 were deeply sedated (RASS-4), no pre–post-test differences were observed in relation to agitation levels. **Conclusions:** The CONECTEM communication intervention outcomes differed between pre- and post-intervention assessments in patients with a Glasgow Coma Scale score of 15–9 regarding pain. These findings are consistent with a potential benefit of the CONECTEM communication intervention, although further studies using designs that allow for stronger causal inference are needed to assess its impact on the psycho-emotional well-being of critically ill patients.

## 1. Introduction

Communication is the essence of the human condition. We express our emotions, feelings, thoughts, aspirations and beliefs, which define us as unique human beings. People who experience critical illness have a limited ability to communicate [1]. Their fragile health status, dependence on mechanical devices to breathe or to replace vital functions, reduced level of consciousness, sedation, or pain hinder their ability to express themselves [2,3]. Ineffective communication leads to frustration, misinterpretation of messages, and, consequently, a reduction in the quality of nursing care provided to the critically ill patient [4,5,6]. For several years, different authors and experts in language and communication have introduced augmentative and alternative communication (AAC) in ICUs to improve effective communication between nurses and patients [7,8,9]. To this end, nursing staff have been trained to implement AAC with critically ill patients, increasing patients’ communication capacity and promoting improvements in nursing care by identifying immediate needs and concerns and enabling more accurate management of pain or symptoms associated with critical illness [10]. Thus, low-technology AAC strategies—such as posters depicting emotions and needs, boards with icons representing basic care (hygiene, nutrition, and comfort), or symptoms such as pain, thirst, and itching—have been better accepted in nurse–patient communication than high-technology AAC (e.g., iPads or specific applications) because of the learning difficulty, lack of time, and workload [10,11]. In addition, the literature indicates that critically ill patients have difficulty handling touch screens because of frailty or weakness, as well as possible visual limitations, as electronic devices are often small [8,12]. Nevertheless, sensory ICUs with touch screens are currently being developed, which could improve this interaction [11,13]. Despite this, training in AAC remains limited, and common barriers persist due to lack of institutional engagement, scarcity of specialists, and difficulties implementing patient-centered models [14]. However, recent reviews highlight that integrating AAC with humanistic practices, ongoing training, and cultural adaptation are key to improving communication in the ICU [15].

Patients admitted to intensive care units (ICUs) face a complex clinical situation that compromises both their physical status and psycho-emotional well-being. The literature shows that psycho-emotional disorders are highly prevalent in this context, significantly affecting the patient’s experience and the quality of care provided [16]. The most common include pain, anxiety, and stressor-related symptoms associated with the ICU experience. Pain affects approximately 70–89% of patients in critically ill patients (ICUs) [17], while anxiety occurs in 30–60% of this population and is often exacerbated by factors such as isolation, invasive procedures, and inadequate communication [15]. In addition, exposure to the ICU environment can precipitate symptoms of post-traumatic stress, such as intrusive memories and avoidance behaviors [18]. These symptoms can progress to post-traumatic stress disorder (PTSD), which affects 20–25% of ICU survivors, with significant long-term consequences for both mental health and overall quality of life [19]. The prevalence of these psycho-emotional effects underscores the critical need for comprehensive assessment and multidisciplinary interventions aimed at mitigating the immediate and long-term psychological sequelae in critically ill patients.

Pain, defined as an unpleasant sensory and emotional experience associated with actual or potential tissue damage [20], is a subjective experience that varies according to individual tolerance thresholds and has multiple dimensions: sensory, emotional, cognitive, psychological, and behavioral. Invasive techniques (tubes, catheters, and mechanical ventilation), the inflammatory process of disease, and immobility are examples of painful processes in the daily experience of the critically ill patient [17,21]. Anxiety is associated with irritating or uncomfortable stimuli and with the perception of threat. The patient’s response depends on the level of interaction with the environment: an anxious, sedated patient may exhibit behaviors such as biting the tube and an increasing heart rate and blood pressure, whereas a conscious patient will express anxiety through crying, complaints, or movements [22]. PTSD, a multifactorial phenomenon, is influenced by psycho-emotional factors, the traumatic event, personal coping, social support, and physical recovery. In critically ill patients, the development of PTSD is related to the actual memory of negative experiences and/or to illusory memories that many sedated patients experience in the medium or long term [23].

Improving communication between nurses and critically ill patients not only optimizes the quality of care but also helps reduce negative psycho-emotional effects such as anxiety, fear, and stress, promoting comfort and patient safety [24,25,26,27]. It is noteworthy that as early as 2010, the Joint Commission had already recognized effective communication as a quality standard with a direct impact on the safety of critically ill patients [28]. The ability to prioritize the patient’s needs is of paramount importance in order to ensure that patient-centered care is provided and positive health outcomes are achieved. Effective communication is therefore crucial to the improvement and prognosis of the critically ill patient [29]. In this context, the evidence supports the implementation of structured communication interventions and conducting rigorous studies to evaluate their impact on the emotional health of critically ill patients.

The primary aim of this study is to evaluate the effectiveness of a communication intervention (CONECTEM) applied to critically ill patients admitted to the ICU in relation to improving psycho-emotional effects (pain, anxiety, and stressor impact).

## 2. Materials and Methods

The study employed a quasi-experimental design with two analysis groups, intervention group (IG) and control group (CG), following a pre–post-test model. In the IG, the communication intervention (CONECTEM) was implemented during nurse–patient communicative interactions, whereas the CG received usual communication practices. The study was conducted and is reported in accordance with the CONSORT 2010 guidelines.

### 2.1. Setting and Sample

The study was conducted in two intensive care units (ICUs) at Vall d’Hebron Hospital, a tertiary public hospital in the province of Barcelona (Catalonia), north-eastern Spain. To avoid contamination, ICU 1 was assigned to the IG and ICU 2 to the CG. ICU 1 and ICU 2 were chosen because they are highly comparable in terms of their structure, care protocols, nurse-to-patient ratios, staff experience, visitation/noise policies, patient case-mix, and catchment area. Specialized ICUs (e.g., trauma, cardiac surgery, pediatric, and burns) were excluded to reduce heterogeneity and unit-level confounding. The sample comprised 180 nurse–patient interactions stratified according to patients’ level of consciousness, assessed using the Glasgow Coma Scale, among critically ill patients admitted to the two ICUs where the study was conducted. There were 61 interactions involving patients with a Glasgow score of 15, 50 interactions involving patients with Glasgow scores of 14–9, and 69 interactions involving patients with Glasgow scores <9. In total, 180 communicative interactions and 111 critically ill patients participated, distributed across the two study groups: 66 patients in the IG and 45 patients in the CG. Patients were included consecutively according to the inclusion/exclusion criteria. Sample size was calculated assuming an alpha risk of 0.05 and 80% power to detect a 25% difference between study groups, allowing for an estimated 10% loss.

Inclusion criteria were critically ill patients admitted to the ICUs of the University Hospital where the study was conducted, who agreed, either personally or via family proxy, to participate in the study after being informed in writing. Exclusion criteria were patients who did not understand Catalan or Spanish, those with an ICU stay <24 h, and/or those with a pre-existing diagnosis of severe psychiatric disorder prior to ICU admission, such as psychotic disorder, schizophrenia, or dementia. Other mental health conditions not classified as severe psychiatric disorders (e.g., anxiety or mild depression) were included.

### 2.2. Data Collection Tools and Methods

#### 2.2.1. Intervention

**Figure 1:**
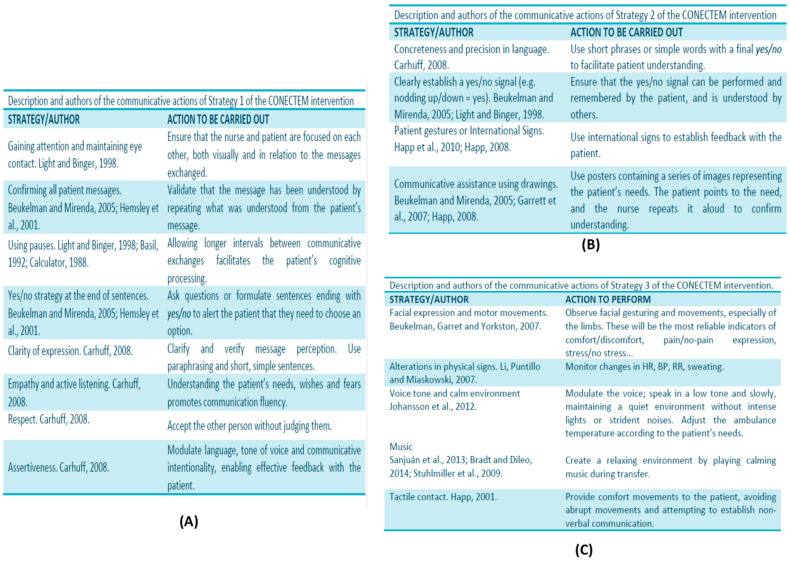
Multicomponent communicative strategies CONECTEM. (**A**) Description of the communicative strategies 1 (Glasgow 15), (**B**) description of the communicative strategies 2 (Glasgow 14–9), (**C**) description of the communicative strategies 2. Prats-Arimon M, (2017) [33].

The CONECTEM communication intervention was based on basic communication skills (BCS) and low-technology-assisted augmentative and alternative communication (AAC), and it addressed four standard communication goals: (1) patients’ needs, (2) information about their health status, (3) communicative elements of socialization, and (4) expression of feelings [30]. The intervention was divided into three communication strategies according to patients’ Glasgow score. Communication strategy 1 was applied to patients with Glasgow 15. Its indicators for effective communication were the following: maintaining eye contact, confirming the patient’s messages, pausing, using yes/no at the end of sentences, clarity of language expression, adopting an empathic attitude and active listening, being respectful, and being assertive. Communication strategy 2 was applied to patients with Glasgow 14–9. Its indicators for effective communication were the following: very concrete and precise language, agreeing using a signal for yes and no, using gestures from the international dictionary, and using posters of needs and emotions and/or a magnetic board with icon magnets related to the four standard communication goals. Communication strategy 3 was applied to patients with Glasgow < 9. Its indicators were the following: observation of facial expression and motor movement, observation of alterations in physical signs, voice tone, promoting a relaxed environmental setting, and using music therapy. Each strategy was validated by a panel of experts, composed of six intensive care and emergency professionals, two mental health specialists, and one communication specialist, who evaluated the communication actions of each strategy on a 4-point Likert scale (1 = not at all appropriate, 4 = very appropriate). All actions were scored as either fairly or very appropriate (mean of 3.8/4) [31]. The strategies were based on the AAC model for critically ill patients proposed by Happ (2008) [32] (see Figure 1).

#### 2.2.2. Procedure

The nurses who participated in the study were required to meet the following criteria in order to contribute to the data collection: work more than 16 h in one of the two ICUs where the study was conducted, have no prior knowledge of AAC, and have at least 2 years of work experience in the ICU. Prior to implementation of the CONECTEM communication intervention, accredited training (University of Barcelona) was provided to the ICU nurses involved in ICU1 (the IG) of the study. The training lasted 5 h and comprised both theoretical teaching and practical workshops and role-playing to acquire communication skills. After training, nurses were assessed to ensure they had acquired the necessary knowledge and skills to implement the intervention. The nurses working in ICU 2, who were part of the CG, did not receive any training in communication skills. As a result, they relied solely on their own communication skills.

**Figure 2:**
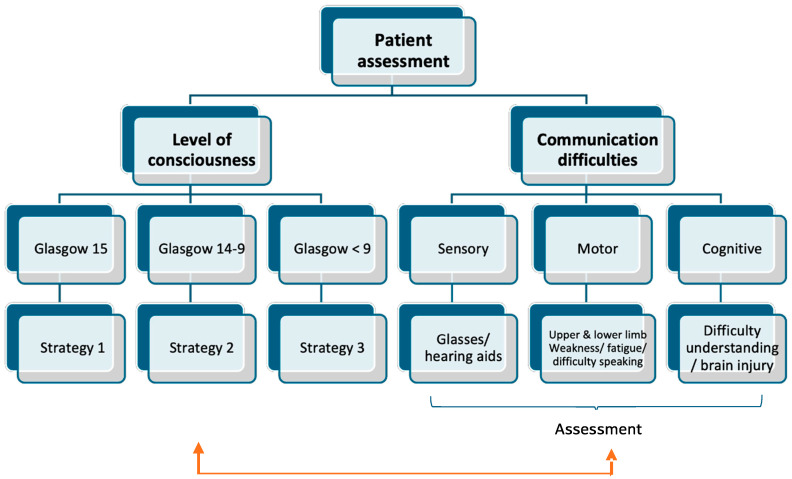
Assessment of communicative difficulties for choosing adequate strategies. Arrow: Selecting the appropriate strategy.

Data were collected by nurses in the two ICUs where the study was conducted between January 2020 and September 2022. When the patient was admitted to the unit, the patient or their family was informed about the purpose of the study. Once participation was accepted voluntarily and inclusion/exclusion criteria were met, patients were recruited consecutively. Severe psychiatric disorders were identified through review of the patients’ documented clinical history. At the initial assessment, an ad hoc questionnaire was used to collect sociodemographic variables (sex, age, educational level, marital status, and provenance), health status variables (type of pathology, endotracheal tube [ETT] carrier), level of consciousness according to the Glasgow scale, and communication difficulties at the sensory (visual, auditory), motor (generalized fatigue, right hemiparesis, facial paralysis, paraplegia, tetraplegia, and dysarthria), and cognitive levels (attention deficit, comprehension deficit, expressive aphasia, receptive aphasia, memory loss, and illusory memory); see Figure 2.

To assess the patient’s psycho-emotional status, the following variables were considered:

Pain: According to the Glasgow score, pain was measured using different scales. Glasgow > 9: the Visual Analog Scale (VAS) [34], consisting of a horizontal line with a score from 0 to 10. The patient is asked to rate their pain along the line, where 0 indicates “I have no pain” and 10 indicates “I have unbearable pain”. Glasgow ≤ 9: the validated Spanish adaptation of Behavior Pain Scale (BPS) [35], which consists of five items evaluating facial musculature, calmness, adaptation to mechanical ventilation, muscle tone, and comfort. Each item is scored from 0 to 2, yielding a total score ranging from 0 (no pain) to 10 (maximum pain). Scores of 1–3 indicate mild to moderate pain, 4–6 moderate to severe pain, and >6 very intense pain.

Anxiety: Glasgow > 9: Spanish short version of the State-Trait Anxiety Inventory (STAI) [36], consisting of 6 items: three items indicating the presence of anxiety (e.g., “I feel distressed,” “I feel nervous,” and “I am worried”) and three items indicating the absence of anxiety (e.g., “I feel comfortable,” “I feel at ease,” and “I feel fine at the moment”). Patients report their current feelings by selecting the items that best reflect their state, and responses were categorized as anxiety present or anxiety absent.

Level of agitation and sedation: The RASS scale was utilized for patients with a Glasgow score < 9 who were connected to mechanical ventilation. The scale comprises nine items ranging from −5 to 4, with the purpose of evaluating the level of agitation and sedation in patients on mechanical ventilation. The range of −5 to −1 on the scale indicates the level of sedation required, with lower values denoting a milder sedation. The value 0 indicates a state of alert and calm; the values from 1 to 4 indicate an increasing level of agitation. The numerical value of the scale corresponds to the intensity of the emotion; for example, number 1 on the scale indicates restlessness, while number 4 indicates combativeness [37].

Post-traumatic stress symptoms: If the patient had a Glasgow > 9, this was measured using the Impact of Event Scale (IES-15) [38], which comprises 15 items: 6 measure intrusion, 8 avoidance, and 1 hyperarousal. The score for each item is measured from 0 to 5, where 0 = never, 1 = sometimes, 3 = often, and 5 = frequently. Based on the total score, PTSD symptoms are classified as mild ≤ 8, moderate 9–19, or severe > 19.

Once baseline had been assessed, nurses in the IG (trained in basic communication techniques (BCS) and AAC) delivered the communication intervention to the patient at least once per shift during 72 h. The intervention had a minimum duration of 20–30 min. Nurses in the CG conducted communicative interactions using usual methods. Post-intervention, psycho-emotional variables were reassessed: pain, anxiety and PTSD symptoms. Data were collected by the research team in an SPSS Standard Edition 22 database.

### 2.3. Data Analysis

An SPSS database was used, in which three communication strategies were applied to 111 patients according to their Glasgow Coma Scale score for a total of 180 strategies analyzed. The communicative strategy was considered the primary unit of analysis rather than the patient. Outcomes were assessed immediately before and after each application of a communication strategy. Individual patients could contribute more than one observation, either through repeated applications of the same strategy across multiple days or through exposure to different strategies. Given that multiple observations could originate from the same patient, results should be interpreted as descriptive and exploratory. No assumptions of statistical independence at the patient level are made.

A descriptive analysis of patients’ baseline characteristics was performed between intervention and control groups, assessing homogeneity using appropriate comparison tests according to the nature of the variables (*t*-test for quantitative variables and chi-square for qualitative variables). The analysis was also repeated by communication strategy groups according to Glasgow score.

For the statistical analysis, pre–post patterns in pain, anxiety, and post-traumatic stress-related behaviors were examined using repeated-measures comparisons for descriptive purposes, and interactions were explored descriptively to examine whether observed changes differed across strategies. Given the quasi-experimental design with a single ICU per group, clustering at the ICU level could not be modeled, and *p*-values are reported for descriptive reference only. Emphasis is placed on observed patterns rather than confirmatory inference. For dichotomous variables, pre–post changes were analyzed using McNemar’s test and differences in the proportion of change across groups.

Pearson’s correlation was used to examine relationships between numerical variables. To explore associations between the studied variables and sociodemographic characteristics (sex, age, and educational level) in the intervention group, the Mann–Whitney U test or Kruskal–Wallis test was applied. Data were analyzed with the PASW-18 statistical package, and *p*-values < 0.05 were considered statistically significant.

## 3. Results

**Figure 3:**
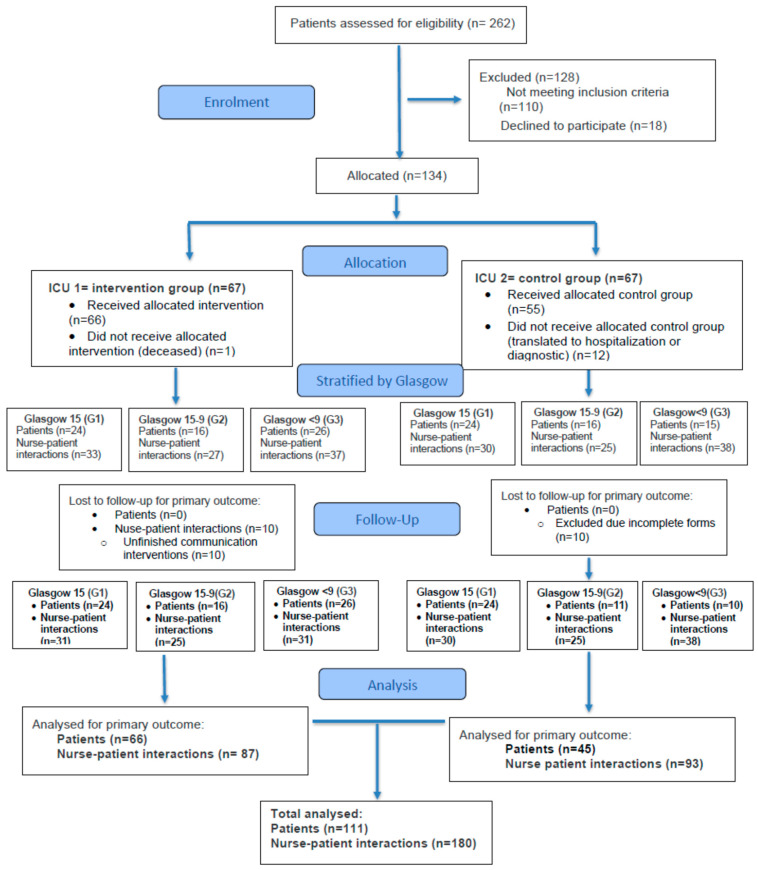
Flow diagram of patients and nurse–patient interactions in the study.

A total of 262 patients were assessed for eligibility, of whom 128 were excluded (116 did not meet inclusion criteria and 21 declined participation). The remaining 134 patients were allocated to either the IG ICU (n = 67) or the GC ICU (n = 67). In the intervention group, no patients were lost to follow-up, although 10 nurse–patient interactions were excluded due to incomplete communication interventions. In the control group, patient loss occurred during follow-up due to transfers to other hospital units or diagnostic procedures, and 10 patients were excluded because of incomplete baseline questionnaires. Consequently, 66 patients in the intervention group and 45 in the control group were included in the final analysis, comprising a total of 111 patients who generated 180 nurse–patient interactions. As multiple interactions could be recorded for the same patient across different days or applications of communication strategies, the number of observations exceeded the number of patients; these interactions were considered for descriptive purposes. Patients were further stratified according to Glasgow Coma Scale scores into three groups: GCS 15, GCS 9–14, and GCS < 9. See Figure 3.

### 3.1. Descriptive Characteristics of Critically Ill Patients

Of the 111 patients included, 61.3% (n = 68) were men. The age groups most frequently represented were 31–50 years at 17% (n = 19) and 51–70 years at 66.1% (n = 73). Only 15.3% (n = 17) of the sample were older than 70 years, and 1.8% (n = 2) were younger than 30 years. Regarding marital status, 65% (n = 72) of critically ill patients were married or partnered, 12.6% (n = 14) were divorced, and 14.4% (n = 16) were single. Approximately 18.9% (n = 21) of patients had no children. The most prevalent pathology was respiratory at 58.7% (n = 65), followed by cardiac at 24.8% (n = 27), and neurological at 9.9% (n = 11). Regarding communication limitations in patients, 10.8% (n = 12) had hearing limitations, 9% (n = 10) had visual limitations, and 50.5% (n = 56) wore glasses. Approximately 4.5% (n = 5) of critically ill patients had cognitive limitations, and 19.8% (n = 22) had generalized fatigue. At the time of assessment, 20.7% (n = 23) of patients were receiving sedation, and 10.8% (n = 12) were receiving neuromuscular relaxation and sedation. Approximately 38.8% (n = 58) of patients were unconscious or had a low level of consciousness (Glasgow < 9), 33.9% (n = 61) had a medium level of consciousness or were disoriented, while 33.9% (n = 61) were conscious and oriented (Glasgow 15). Statistical tests showed no significant baseline differences between the patients in the CG and the IG (See Table 1). Similarly, when patient characteristics were examined descriptively across Glasgow Coma Scale categories (15, 9–14, and <9), no clear differences were observed.

### 3.2. Baseline Levels of Pain, Anxiety, and Post-Traumatic Stress Symptoms in CG and IG According to Glasgow Score (Group 1 = Glasgow 15; Group 2 = Glasgow 14–9; and Group 3 = Glasgow < 9)

At pre-test, the three psycho-emotional variables (pain, anxiety, and post-traumatic stress symptoms) were examined descriptively across Glasgow Coma Scale categories and between the CG and IG. No clear differences were observed in the baseline values of the three psycho-emotional variables across Glasgow levels or between groups.

As shown in Table 2, pain levels in both the CG and IG among patients in Group 1 (Glasgow 15) and Group 2 (Glasgow 14–9) were generally low, with a mean VAS score < 3, corresponding to mild pain. Overall, 35.1% (n = 39) of patients reported no pain, 44% (n = 50) mild pain, 18% (n = 19) moderate pain, and 1.8% (n = 2) reported severe pain. The maximum VAS pain score was 7 in both groups. By contrast, patients in Group 3 (Glasgow < 9) showed low pain on the BPS scale, with a mean score < 1 and a maximum score of 5 out of 0 (indicating no observable pain) were recorded in 97% (n = 67) of nurse-patient interactionsin this group.

Regarding anxiety, both the CG and IG among patients with a level of consciousness (Glasgow 15–9) reported the presence of anxiety symptoms (“I feel nervous”) on the STAI scale) at the pre-test, with proportions of 56.4% (n = 30) CG vs. 50% (n = 28) IG. Concerning post-traumatic stress symptoms, both the CG and IG in Glasgow Groups 1 and 2 presented elevated symptom levels at baseline (mean > 19 points on the IES scale). Overall, 19.8% (n = 22) reported moderate stress symptoms and 68% (n = 46) severe stress symptoms. In Group 3, both CG and IG were deeply sedated and did not display signs of agitation, with mean RASS scores of [−4.00 (SD 1.21) CG vs. −4.14 (SD 1.18) IG].

### 3.3. Pre–Post-Test Comparison Between CG and IG Nurse–Patient Interactions for the Three Analyzed Variables According to Glasgow Score

Table 3 presents pre–post changes in pain levels and post-traumatic stress symptoms following the application of communication strategies. In the IG, lower pain scores were observed at the post-test compared with the pre-test among patients with preserved or partially preserved levels of consciousness (Glasgow Coma Scale scores of 15 and 9–14), both in patients with Glasgow 15 [MD 1.13 (±1.36) vs. 0.71 (±1.07)] and in patients with Glasgow (14–9) [MD 2.64 (±2.10) vs. 1.68 (±2.04)]. Post-intervention, 89.3% (n = 50) of patients in the IG reported no pain or mild pain, whereas only 10.4% (n = 6) reported moderate pain. By contrast, pain levels in the CG showed no significant differences in pain, either in patients with Glasgow 15 (*p* = 0.614) or in patients with Glasgow 14–9 (*p* = 0.670).

Regarding the studied variable impact of stressful events (post-traumatic stress symptoms), no pre–post-test differences were observed in either ICU. Nevertheless, in the IG, mean IES- scores showed a downward trend from pre- to post-test. Specifically, in Group 1 (Glasgow 15), mean scores decreased from 24.7 (±15.20) to 22.5 (±14.11), and in Group 2 (Glasgow 14–9), from 30.2 (±13.56) to 27.9 (±11.14). Although these changes suggest a reduction in post-traumatic stress symptoms at post-test, they did not reach conventional thresholds for statistical significance. In the CG, mean IES scores remained stable between the pre- and post-test, as shown in Table 3.

Regarding anxiety, pre–post patterns differed between the IG and CG among communicative interactions involving patients with preserved or partially preserved levels of consciousness stratified by the Glasgow Coma Scale (*p* = 0.007). In the IG, the proportion of communicative interactions in which anxiety was present decreased from 50% (n = 28 communicative interactions) in the pre-test to 27.7% (n = 14 communicative interactions) at post-test. Accordingly, 73.3% (n = 41) of communicative interactions at post-test were associated with the absence of anxiety following the communication intervention. In contrast, in the CG with Glasgow scores between 15 and 9, the proportion of interactions associated with anxiety remained relatively stable over time. Anxiety was present in 56.4% (n = 30) of communicative interactions at pre-test and in 55.0% (n = 30) at the post-test; the proportion of interactions characterized by the absence of anxiety remained similar [43.6% (n = 24) pre-test vs. 45.5% (n = 25) post-test]. Statistical comparisons indicated that the magnitude of pre–post change in anxiety differed between ICUs (Table 4). Given the study design and unit of analysis, these findings should be interpreted as descriptive patterns rather than confirmatory evidence of an intervention effect.

Nurse–patient interactions performed in patients in Group 3 (Glasgow < 9) were characterized by consistently low scores across all assessed outcome variables at both pre-test and post-test. At pre-test, the mean BPS score was very low in both the IG and CG (MD = 0.15 and 0.00, respectively), with a maximum observed score of 2. In the post-test, the mean BPS score in both groups was 0.05, also with a maximum of 2. These low scores suggest minimal observable pain responses in this subgroup, meaning they either did not experience observable pain, or pain could not be reliably assessed due to their deep sedation. Similarly, regarding agitation and sedation, patients in this group were deeply sedated at baseline, with a mean RASS score of −4 in both the IG and CG and no noticeable variation over time.

### 3.4. Analysis of Pre–Post-Test Differences in the Studied Variables by Glasgow Group in Relation to Sex and Age

In the IG, pre–post patterns in the studied variables were examined descriptively according to age, sex, and educational level using non-parametric tests. Results showed that, in Group 1 (Glasgow 15), reductions in post-traumatic stress symptoms were larger in women than men (*p* < 0.001), with a mean reduction of 4 points in the IES score at post-test and a maximum score of 18, corresponding to a moderate level of post-traumatic stress symptoms. No other differences were observed according to age or educational level nor in other Glasgow groups or outcome variables. Statistical comparisons are presented for descriptive purposes only.

## 4. Discussion

Critical illness entails a series of conditions that impact the quality of life of the person who suffers it [39]. The life-threatening risk to which the person is exposed shakes all essential dimensions, leading to a situation of vulnerability and fragility. Physical status, dependence on devices and/or drugs, level of consciousness, emotional impact, inability to make decisions, fear, and pain, among other factors, hinder communication capacity, making the ICU experience stressful for the patient [23,40,41]. The literature has extensively shown that critically ill patients present psycho-emotional disorders in the short, medium, and long term [23,42,43]. The most relevant are anxiety, depression, and post-traumatic stress. Several authors agree that the prevalence of psycho-emotional effects derived from critical illness increases in patients who have undergone deep sedation, have more days on mechanical ventilation, and have experienced pain or illusory memory [40,41,44]. In the present study, only 31.5% (n = 35) of the total sample received mechanical ventilation and required sedation and/or relaxation. Nevertheless, the baseline prevalence of anxiety among non-sedated patients with a Glasgow score > 9 was comparable (50–55%) to that reported in studies including predominantly mechanically ventilated patients [41,45]. Post-traumatic stress symptoms were notable, with mean IES scores between 20 and 29 points in patients with Glasgow scores between 9 and 15. As widely described in the literature, admission to and/or stay in the ICU is generally perceived as a highly stressful experience for both patients and relatives [39,46,47]. When compared with previous studies, the prevalence of post-traumatic stress symptoms in our sample appeared higher than that reported elsewhere, with 19.8% (n = 22) of patients showing moderate stress symptoms and 68% (n = 46) severe stress symptoms, whereas other studies have reported prevalences of approximately 13% for high and 17.8% for moderate PTSD symptoms [48]. In this context, it may be hypothesized that non-sedated patients, by virtue of having greater awareness and cognitive capacity to understand their clinical situation, may experience a higher perceived impact of ICU-related stressors.

Pain levels in patients with a level of consciousness > 9 generally ranged from mild to moderate, with the highest VAS score observed being 7. Pain is commonly reported among critically ill patients and may be influenced by invasive procedures, immobility, and limitations in nurse–patient communication, all of which can hinder optimal pain management [49,50]. By contrast, 97% of mechanically ventilated and sedated patients in the sample did not show behavioral indicators of pain, with a mean BPS score of 0.07; only one patient presented a BPS score of 5, corresponding to moderate pain.

However, when interpreting these findings, it should be noted that RASS scores in this patient group were predominantly −4 or −5, indicating deep sedation. Therefore, it is possible that the level of sedation limited the accurate behavioral assessment of pain in these patients. This pattern may be partly attributable to the high proportion of patients with severe respiratory pathology, particularly acute exacerbations of COVID-19, who required deep sedation as part of their clinical management. Another potential explanation could be that the absence of pain and the deep state of sedation presented by patients in this group at baseline leads us to consider the possibility that the lack of significant differences in this group could be due to the assessment tools (BPS/RASS scales) lacking the necessary sensitivity to capture changes in patients with a RASS score of −4 rather than the intervention necessarily being ineffective. This phenomenon is known in the field of statistics as the ‘floor effect’.

### 4.1. Effective Communication and Psycho-Emotional Effects

Effective communication is a quality standard in nursing care for critically ill patients. The need to express basic needs such as thirst; repositioning; feeling cold; emotional needs (fear, anger, anxiety, and pain); informational needs about the illness; or socialization needs (thank you, good morning, and please) constitutes four standard communication goals in the critically ill patient [11,51]. The communication difficulties experienced by critically ill patients limit their ability to communicate, producing frustration and misunderstandings in nurse–patient interactions, which affects nursing care [5,52]. Sanderson et al. (2019) indicate that 35% of critically ill patients had difficulties communicating [3]. The communication strategies were applied to interactions involving patients with different levels of complexity related to sensory limitations, cognitive limitations, or motor limitations. For this reason, nurses assessed the patients’ communication difficulties and applied the most appropriate communication strategy, also considering the level of consciousness. Clear instructions were used, such as maintaining eye contact, repeating the patient’s message, asking short yes/no questions, and establishing a confirmation signal for yes and no, in addition to applying basic communication skills such as empathy and active listening. Different large-format posters with emotions and needs were also used. Patients with sufficient upper-limb mobility had access to a magnetic board with different icons related to the four standard communication goals to enable expression. The results of the present study suggest that the use of low-learning-burden AAC strategies may be associated with lower levels of pain and anxiety in patients with a Glasgow Coma Scale score between 9 and 15. These observations are broadly consistent with the previous literature in which authors have highlighted that effective communication with critically ill patients may support improved pain management and psycho-emotional outcomes, thereby potentially contributing to better nursing care quality and patient well-being [19,30,52,53]. In this context, Happ et al. (2014) reported an association between the implementation of low-tech AAC strategies and reduced pain levels, which resembles the pattern observed in the present study [7]. However, pain management is sometimes influenced by nurses’ motivation to use pictograms and/or their willingness to attempt to communicate with the patient [54,55]. The literature recommends multidisciplinary interventions to address the patient comprehensively, considering both physical and psycho-emotional symptoms [56]. Accordingly, interventions based on non-verbal communication (tactile contact, a quiet environment without noise, and visual stimulation) and non-pharmacological measures such as music therapy and aromatherapy have been implemented to reduce psycho-emotional effects such as anxiety or pain, with significant improvements in these symptoms [57,58,59].

It has been shown that light sedation improves outcomes in critically ill patients by reducing the length of stay and shortening extubation time [60,61]. However, current clinical guidelines agree that deep sedation may be necessary in specific clinical contexts, such as severe respiratory failure, acute ARDS, or complex exacerbations. Although it is true that formal indicators of patient severity were not collected in the present study, these pathologies were common during the post-peak COVID study period in 2020 [62]. The effectiveness of communication in analgosedated patients for the management of pain and/or anxiety has been positive in patients with RASS −1/1 [25,63]. In deeply sedated or non-communicative critically ill patients, positive RASS scores indicate clinically relevant agitation and restlessness. Although the RASS was not originally designed to assess anxiety or post-traumatic stress symptoms, higher scores have been associated with delirium, acute brain dysfunction, and distress-related behavioral states in the ICU [64]. In this context, agitation may serve as an observable behavioral marker of underlying psychological distress when patient self-report is not feasible. These findings support the clinical relevance of RASS as a complementary tool for identifying distress in critically ill patients [65].

Despite this, recent studies have reinforced the need to implement interventions to address psycho-emotional aspects derived from critical illness that affect quality of life once patients are discharged from the ICU [4,42,56]. Anxiety, depression and post-traumatic stress symptoms are the main post-ICU disorders experienced by these patients [19,42]. The use of AAC to achieve effective nurse–patient communication may facilitate a comprehensive approach to patient care. As experts in the field have emphasized, implementing these communication techniques requires prior nurse training, appropriate and necessary resources, and the integration of effective communication into the nursing care plan [10,53,66]. In Spain, the exploration of the impact of effective communication on the well-being of critically ill patients considering all their dimensions has only recently begun [39,67], which calls for in-depth study of the implementation of AAC techniques in our country [39,42]. The results of this study provide an initial descriptive exploration of patterns observed in the application of AAC in relation to the psycho-emotional outcomes in critically ill patients without implying causal inference.

### 4.2. Study Strengths and Limitations

The main limitations of this study are related to the study design and the characteristics of the patient sample, such as the following: the inclusion of patients with a Glasgow Coma Scale score < 9 without defining a restricted RASS range. Second, the use of the RASS as a proxy for psychological distress in non-communicative patients represents an important limitation, as it does not directly measure subjective constructs such as anxiety or post-traumatic stress symptoms and should be interpreted as an indicator of observable agitation rather than psychological distress. Third, the study design involved a single ICU per condition, and analyses were performed at the level of nurse–patient interactions. While repeated-measures ANOVA was applied to explore pre–post patterns, the lack of multiple clusters limits the ability to separate unit-level effects from intervention-related patterns. As such, statistical results, including *p*-values, should be interpreted as descriptive observations rather than confirmatory evidence. Fourth, while baseline characteristics between groups were generally similar, the study did not use mixed-effects modeling to account for intra-patient correlations arising from multiple interactions, which may limit the modeling of intra-subject variability. Finally, the study did not assess the longitudinal evolution of anxiety or post-traumatic stress symptoms after ICU discharge, and patient characteristics or sedative practices may have influenced the number and type of interactions available for analysis.

The study provides an innovative approach by exploring patterns in psycho-emotional outcomes observed during nurse–patient interactions in critically ill patients during ICU stay, highlighting the role of communication strategies in contexts of deep sedation. By describing these patterns, the study sheds light on a scarcely explored area and may inform future research aimed at improving the quality of care and patient experience. Longitudinal follow-up after ICU discharge could help further characterize associations between communication strategies and psycho-emotional outcomes over time.

## 5. Conclusions

The CONECTEM communication intervention based on basic and assisted communication techniques was applied in nurse–patient interactions with critically ill patients with Glasgow scores of 15–9. Patterns observed in these interactions suggest reductions in reported pain and anxiety in critically ill patients, as well as decreases in post-traumatic stress-related behaviors, although the latter did not reach conventional thresholds for statistical significance. These descriptive results highlight the potential role of structured communication strategies in supporting patient-centered care in the ICU. Further studies in Spanish ICUs are recommended to further explore associations between communication strategies and the psycho-emotional outcomes experienced by critically ill patients, including follow-up after ICU discharge.

## Figures and Tables

**Table 1:** Baseline of sociodemographic and health patient characteristics.

Variable		IG (n = 66)	CG (n = 45)	Total (111)	*p*-Value
Gender	Female	**25** (38.5%) [26.7; 51.4]	**19** (40.9%) [26.3; 56.8]	**44** (39.4%) [30.2; 49.3]	0.797 ^2^
	Male	**40** (61.5%) [48.6; 73.3]	**27** (59.1%) [43.2; 73.7]	**67** (60.6%) [50.7; 69.8]	
Age groups	18–30	**2** (3.1%) [0.4; 10.7]	**0** (0%) [0.0; 8.0]	**2** (1.8%) [0.2; 6.5]	0.560 ^2^
	31–50	**11** (16.9%) [8.8; 28.3]	**9** (18.2%) [8.2; 32.7]	**19** (17.4%) [10.8; 25.9]	
	51–70	**44** (67.7%) [54.9; 78.8]	**29** (63.6%) [47.8; 77.6]	**73** (66.1%) [56.4; 74.9]	
	71–90	**9** (12.3%) [5.5; 22.8]	**8** (18.2%) [8.2; 32.7]	**17** (15.3%) [8.6; 22.7]	
Studies level	Primary studies	**27** (41.6%) [26.7; 51.4]	**22** (49.1%) [30.4; 61.2]	**49** (44.1%) [31.9; 51.1]	0.662 ^5^
	Secondary studies	**30** (46.1%) [31.6; 54.3]	**19** (36.3%) [18.2; 44.4]	**49** (44.1%) [43.8; 57.8]	
	University studies	**7** (10.8%) [4.4; 20.9]	**4** (9.1%) [2.5; 21.7]	**11** (10.1%) [5.1; 17.3]	
	Postgraduate studies	**0** (0%) [0.0; 5.5]	**2** (4.5%) [0.6; 15.5]	**2** (1.8%) [0.2; 6.5]	
Marital status	Single	**12** (18.5%) [9.9; 30.0]	**3** (6.8%) [1.4; 18.7]	**15** (13.8%) [7.9; 21.7]	0.319 ^5^
	Married	**43** (66.5%) [53.6; 70.6]	**29** (66.0%) [41.0; 71.7]	**72** (65.0%) [48.0; 67.2]	
	Divorced	**6** (9.2%) [3.5; 19.0]	**8** (18.2%) [8.2; 32.7]	**14** (12.8%) [7.2; 20.6]	
	Widower	**4** (6.2%) [1.7; 15.0]	**4** (9.1%) [2.5; 21.7]	**8** (7.3%) [3.2; 14.0]	
Nationality	Spain	**55** (84.6%) [73.5; 92.4]	**38** (86.4%) [72.6; 94.8]	**93** (85.3%) [77.3; 91.4]	0.928 ^5^
	Europe	**3** (1.5%) [0.0; 8.3]	**3** (0%) [0.0; 8.0]	**6** (0.9%) [0.0; 5.0]	
	South America	**3** (1.5%) [0.0; 8.3]	**2** (0%) [0.0; 8.0]	**5** (0.9%) [0.0; 5.0]	
	Argentine	**0** (0%) [0.0; 5.5]	**1** (2.3%) [0.1; 12.0]	**1** (0.9%) [0.0; 5.0]	
	Asia	**1** (0%) [0.0; 5.5]	**1** (2.3%) [0.1; 12.0]	**2**(.9%) [0.0; 5.0]	
	Africa	**1** (1.5%) [0.0; 8.3]	**1** (2.3%) [0.1; 12.0]	**2** (1.8%) [0.2; 6.5]	
Types of pathology	Cardiac	**16** (24.6%) [14.8; 36.9]	**11** (25%) [13.2; 40.3]	**27** (24.8%) [17.0; 34.0]	0.089 ^5^
	Respiratory	**43** (66.2%) [53.4; 77.4]	**22** (47.7%) [32.5; 63.3]	**65** (58.7%) [48.9; 68.1]	
	Neurologic	**5** (6.2%) [1.7; 15.0]	**6** (13.6%) [5.2; 27.4]	**11** (9.9%) [4.5; 16.2]	
	Shock	**0** (0%) [0.0; 5.5]	**2** (4.5%) [0.6; 15.5]	**2** (1.8%) [0.2; 6.5]	
	Psychiatric	**2** (3.1%) [0.4; 10.7]	**4** (9.1%) [2.5; 21.7]	**6** (5.5%) [2.0; 11.6]	
Auditive limitations	No	**57** (87.7%) [77.2; 94.5]	**38** (86.4%) [72.6; 94.8]	**95** (87.2%) [79.4; 92.8]	0.525 ^5^
	Yes	**6** (9.2%) [3.5; 19.0]	**6** (13.6%) [5.2; 27.4]	**12** (11%) [5.8; 18.4]	
	Wears hearing aids	**2** (3.1%) [0.4; 10.7]	**0** (0%) [0.0; 8.0]	**2** (1.8%) [0.2; 6.5]	
Visual limitations	No	**26** (40%) [28.0; 52.9]	**18** (40.9%) [26.3; 56.8]	**44** (40.4%) [31.1; 50.2]	0.778 ^2^
	Yes	**5** (7.7%) [2.5; 17.0]	**5** (11.4%) [3.8; 24.6]	**10** (9.2%) [4.5; 16.2]	
	Wears glasses	**34** (52.3%) [39.5; 64.9]	**21** (47.7%) [32.5; 63.3]	**55** (50.5%) [40.7; 60.2]	
Cognitive limitations	No	**38** (58.5%) [45.6; 70.6]	**32** (72.7%) [57.2; 85.0]	**70** (64.2%) [54.5; 73.2]	0.098 ^5^
	Attention deficit	**0** (0%) [0.0; 5.5]	**1** (2.3%) [0.1; 12.0]	**1** (0.9%) [0.0; 5.0]	
	Memory loss	**1** (1.5%) [0.0; 8.3]	**0** (0%) [0.0; 8.0]	**1** (0.9%) [0.0; 5.0]	
	Others	**2** (3.1%) [0.4; 10.7]	**0** (0%) [0.0; 8.0]	**2** (1.8%) [0.2; 6.5]	
Motor limitations	No	**29** (44.6%) [32.3; 57.5]	**18** (40.9%) [26.3; 56.8]	**47** (43.1%) [33.7; 53.0]	0.188 ^5^
	Generalized fatigue	**9** (13.8%) [6.5; 24.7]	**12** (27.3%) [15.0; 42.8]	**21** (19.3%) [12.3; 27.9]	
	Others	**2** (3.1%) [0.4; 10.7]	**4** (9.1%) [2.5; 21.7]	**6** (5.5%) [2.0; 11.6]	
Sedation		**19** (29.2%) [18.6; 41.8]	**5** (11.4%) [2.5; 21.7]	**23** (21.1%) [13.9; 30.0]	0.111 ^5^
Sedation and relaxtion		**6** (9.2%) [3.5; 19.0]	**6** (13.6%) [5.2; 27.4]	**12** (11%) [5.8; 18.4]	

^2^: Chi-squared test, arithmetic mean (SD); [95% confidence interval]; median (P25; P75); minimum/maximum; ^5^: Fisher’s exact test.

**Table 2:** Baseline psycho-emotional effects: pain, anxiety, and post-traumatic stress symptoms according to level of consciousness.

	GROUP 1 (Glasgow 15)			GROUP 2 (Glasgow 14–9)			GROUP 3 (Glasgow < 9)		
	CG (n = 30)	IG (n = 31)	*p*-Value	CG (n = 25)	IG (n = 25)	*p*-Value	CG (n = 38)	IG (n = 31)	*p*-Value
VAS-pre	**1.97** (2.06) [1.20; 2.74] **1.50** (0.00; 3.00) 0.00/7.00	**1.54** (1.78) [1.09; 2.00] **1.00** (0.00; 2.0) 0.00/7.00	0.180 ^2^	**1.65** (1.90) [0.67; 2.62] **1.00** (0.00; 3.00) 0.00/6.00	**2.24** (2.06) [1.60; 2.88] **2.00** (0.00; 4.00) 0.00/7.00	0.117 ^2^			
IES-R-pre	**20.00** (16.76) [13.74; 26.26] **15.50** (7.00; 32.00) 0.00/62.00	**22.39** (15.24) [18.49; 26.0] **20.00** (11.00; 32.0) 0.00/62.0	0.088 ^2^	**28.82** (14.62) [21.31; 36.3] **31.00** (15.00; 40.0) 10.00/64.00	**29.64** (14.80) [25.03; 34.2] **31.00** (19.00; 40.0) 0.00/64.00	0.626 ^2^			
STAI-pre PA AA	**43.6% (n13)** 56.4% (n17)	**50% (n30)** 50% (n30)	0.830 ^1^	**43.6% (n7)** 56.4% (n9)	**50% (n21)** 50% (n21)	0.830 ^1^			
BPS-pre							**0.00** (0.00) [0.00] **0.00** (0.00; 0.00) 0.00/0.00	**0.07** (0.60) [−0.07; 0.22] **0.00** (0.00; 0.00) 0.00/5.00	0.268 ^2^
RASS-pre							**−4.00** (1.21) [−4.40; −3.6] **−4.00** (−5.00; −3.0) −5.00/−1.00	**−4.14** (1.18) [−4.43; −3.8] **−4.00** (−5.00; −4.0) −5.00/1.00	0.230 ^2^

IG (intervention group); CG (control group); VAS: Visual Analog Scale; STAI: State-Trait Anxiety Inventory; PA: anxiety present; AA: anxiety absent; IES: Impact of Event Scale; arithmetic mean (SD) [95% confidence interval], median (P25; P75), and minimum/maximum; ^2^: Mann–Whitney U test; and ^1^: Pearson’s chi-square test.

**Table 3:** Pre–post-test difference in pain and stressor impact variables between IG and CG for Glasgow Group 1 and Group 2.

		VAS					
		Pre-Test	Post-Test	*p*-Value	Pre-Test	Post-Test	*p*-Value
GROUP 1 (Glasgow 15)	IG (n = 31)	**1.13** (1.36) **1.00** (0.00; 1.00)	**0.71** (1.07) **0.00** (0.00; 1.00)	**0.008 ^2^** *	**24.71** (13.49) **25.00** (16.00; 35.00)	**22.81** (12.13) **22.00** (15.00; 30.00)	0.207 ^2^
	CG (n = 30)	**1.97** (2.06) **1.50** (0.00; 3.00)	**1.80** (1.92) **1.50** (0.00; 3.00)	0.614 ^2^	**20.00** (16.76) **15.50** (7.00; 32.00)	**20.00** (16.19) **16.00** (7.00; 27.00)	0.859 ^2^
GROUP 2 (Glasgow 14–9)	IG (n = 25)	**2.64** (2.10) **3.00** (1.00; 4.00)	**1.68** (2.04) **1.00** (0.00; 3.00)	**0.008 ^2^** *	**30.20** (15.20) **31.00** (21.00; 38.00)	**27.92** (14.11) **30.00** (19.00; 36.00)	0.358 ^2^
	CG (n = 25)	**1.64** (1.82) **1.00** (0.00; 3.00)	**1.40** (1.58) **1.00** (0.00; 2.00)	0.670 ^2^	**29.48** (13.56) **30.00** (16.00; 40.00)	**29.12** (11.14) **29.00** (19.00; 40.00)	0.966 ^2^

IG (intervention group); CG (control group); VAS: Visual Analog Scale; arithmetic mean (SD); median (P25; P75); ^2^: repeated ANOVA for ranks; and * (significant test).

**Table 4:** Pre–post changes in anxiety (STAI) by intervention group (IG) and control group (CG), stratified by Glasgow Coma Scale.

STAI	Presence of Anxiety		Absence of Anxiety		*p*-Value (Within Group, McNemar)	*p*-Value (Between Groups, χ^2^)
Glasgow (15–9) G1/G2	Pre-Test	Post-Test	Pre-Test	Post-Test		
IG (n = 56 nurse–patient interactions)	50% (n = 28)	27.7% (n = 14)	50% (n = 28)	73.3% (n = 42)	**0.** 007 *	**0.000** *
CG (n = 55 nurse–patient interactions)	56.4% (n = 31)	55% (n = 30)	43.6% (n = 24)	45% (n = 25)	1.000	

IG (intervention group); CG (control group); STAI, State-Trait Anxiety Inventory; cross-tabulation tests: McNemar test (within groups); chi-square test on the dichotomous pre–post change in anxiety (change vs. no change); and * (significant test). Bold indicate statistically significant *p*-values.

## Data Availability

The data that support the findings of this study are available from the corresponding author upon reasonable request.

## Abbreviations

The following abbreviations are used in this manuscript:

ICU	Intensive Care Unit
AAC	Augmentative and Alternative Communication
VAS	Visual Analogic Scale
BPS	Behavioral Pain Scale
IES-R	Impact Event Scale Revised
RASS	Richmon Agitation Sedation Scale
BCS	Basic Communication Skills
STAI	State-Trait Anxiety Inventory
CG	Control Group
IG	Intervention Group
